# Chemical Compounds and Bioactivity of Aqueous Extracts of *Alibertia* spp. in the Control of *Plutella xylostella* L. (Lepidoptera: Plutellidae)

**DOI:** 10.3390/insects8040125

**Published:** 2017-11-22

**Authors:** Lucas L. S. Peres, Ana I. Sobreiro, Irys F. S. Couto, Rosicléia M. Silva, Fabricio F. Pereira, Silvia C. Heredia-Vieira, Claudia A. L. Cardoso, Munir Mauad, Silvana P. Q. Scalon, Sandra S. Verza, Rosilda M. Mussury

**Affiliations:** 1 Laboratory of Insect-Plant Interaction, Faculty of Biological and Environmental Sciences, Federal University of Grande Dourados, Highway Dourados-Itahum, km 12, Dourados 79804-970, Mato Grosso do Sul, Brazil; lucaslsperes@gmail.com (L.L.S.P.); bel_sobreiro@hotmail.com (A.I.S.); irys.ento@gmail.com (I.F.S.C.); rosi_girs@hotmail.com (R.M.S.); 2 Laboratory of Biological Control, Faculty of Biological and Environmental Sciences, Federal University of Grande Dourados, Highway Dourados-Itahum, km 12, Dourados 79804-970, Mato Grosso do Sul, Brazil; fabriciofagundes@ufgd.edu.br; 3 Laboratory of Chemistry, State University of Mato Grosso do Sul, Highway Dourados-Itahum, km 12, Dourados 79804-970, Mato Grosso Do Sul, Brazil; silviacristina_85@hotmail.com (S.C.H.-V.); claudia@uems.br (C.A.L.C.); 4 Laboratory of Vegetables Production, Faculty of Agricultural Sciences, Federal University of Grande Dourados, Highway Dourados-Itahum, km 12, Dourados 79804-970, Mato Grosso Do Sul, Brazil; munirmauad@ufgd.edu.br (M.M.); silvanascalon@ufgd.edu.br (S.P.Q.S.); 5 Laboratory of Social Insects-Pest, Department of Vegetal Production, Phytosanitary Defense, Faculty of Agronomic Sciences, São Paulo State University, Street José Barbosa de Barros, 18610-307, Botucatu, São Paulo 18610-307, Brazil

**Keywords:** *Alibertia edulis*, *Alibertia intermedia*, *Alibertia sessilis*, flavonoid, phenolic compounds, Plutella xylostella

## Abstract

Successive applications of insecticides to control *Plutella xylostella* L. (Lepidoptera: Plutellidae) have resulted in the emergence of resistant populations of this insect. A novel control measure for this target insect could be the use of botanical insecticides derived from plant tissues. Hence, we experimentally tested aqueous extracts of *Alibertia edulis* (Rich.), *Alibertia intermedia* (Mart.), and *Alibertia sessilis* (Vell.) K. Schum. found in the Brazilian savannah in order to investigate their potential to disrupt the life cycle of *P. xylostella*. Aqueous extracts of the leaves of *A. intermedia* and *A. sessilis* negatively affected the development of *P. xylostella* in all stages of the life cycle, prolonging the larval stage and causing mortality in the larval or pupal stages. Treatments with *A. intermedia* and *A. sessilis* extracts caused the lowest fecundity and the number of hatched larvae. The harmful effects of these aqueous extracts on the life cycle of *P. xylostella* may be attributable to the flavonoids and other phenolic compounds present in *A. intermedia* and *A. sessilis*. These aqueous botanical extracts are low in toxicity when compared to non-aqueous pesticides, and may emerge as an effective approach for control of populations of *P. xylostella*.

## 1. Introduction

*Plutella xylostella* (Linnaeus, 1758) (Lepidoptera: Plutellidae), popularly known as the diamondback moth (DBM), is one of the causative agents of brassica damage [1]. Every year, this species causes in excess of $1 billion worth of damage worldwide [2]. This is a consequence of the high feeding rates of this insect during the larval period, which can result in significant crop losses [3].

Pesticide application is still the preferred method of farmers to control damaging insect populations [4]. However, *P. xylostella* has developed resistance to many synthetic insecticides because of its high fertility, tendency towards overlapping generations, genetic plasticity, and, in particular, selection pressure from the use of various insecticides. The application of pesticides, in some years has, nevertheless, been shown to be ineffective in the control of *P. xylostella*, even after 15 to 20 applications during a crop cycle [5,6,7]. Pesticide use is particularly common due to its practicality, speed, and efficiency in population control [4]; however, its use can select more resistant individuals [8], thereby exacerbating the problem. Therefore, a number of studies have focused on the control of this insect, ranging from those involving the genes responsible for the production of saponins [9] and proteins [10], to microbial essays [11] and the use of insecticidal plants [8,12]. The use of botanical insecticides represents an interesting alternative to control insects. These insecticides can be prepared from locally available plants [8], have an advantageous cost/benefit ratio [12], and have low toxicity [10,13,14]. Several plant species have been examined to evaluate their insecticidal effect, since they may offer higher selectivity [6,7], lower toxicity to non-target organisms [4], local availability, low resistance, and low cross-resistance due to their natural complexes [12,13]. Studies conducted by Amoabeng et al. [12], which assessed the cost/benefit ratio of using plants to control insects, have revealed that botanical insecticides might differ in the level of pest control and beneficial cost when compared with conventional insecticides. However, these biopesticides are easily produced from available naturally-growing plants.

Insecticidal plants can inhibit insect feeding, chitin biosynthesis, and oviposition; reduce intestinal motility, fecundity, and longevity; interfere with ecdysone synthesis; deform both pupae and adults; sterilize individuals; and kill both immature and adult individuals [15,16].

There have been numerous studies on the insecticidal potential of plants [17,18,19]. Plants in the Rubiaceae family, widely distributed in the Brazilian savannah, have insecticidal properties in addition to their economic relevance. The Rubiaceae family is characterized by the presence of several classes of substances, including phenolic compounds [20,21]. Flavonoids and phenolic and polyphenol acids are among the most prominent [22], in addition to caffeic acids in the *Alibertia* genus [23,24]. Furthermore, the presence of iridoids [25], alkaloids [26], anthraquinones [27], lignones [28], flavonoids, phenolic derivatives, triterpenes, and diterpenes [20] have all been reported in the literature.

The leaf and root extracts of *Palicourea* St. Hill have been demonstrated to be toxic to adults of *Aetelion* sp. (Hemiptera: Aetalionidae), possibly due to the presence of monofluoroacetic acid that interferes with the Krebs cycle and decreases cellular respiration [29]. *Palicourea marcgravii* St. Hill. has been shown to be efficient in causing mortality of *Aphis spiraecola* Patch. (Hemiptera: Aphididae). This is probably attributable to the high concentration of monofluoroacetic acid and/or fluoroacetate that, acting synergistically with other compounds, induces a greater toxicity [30]. *Catunaregam spinosa* (Thunb.) Tirveng. contains triterpenoid saponins with antifeedant activities against *P. xylostella* [31].

Other botanical families that also show insecticidal activity are the following: Meliaceae, Annonaceae, Asteraceae, Canellaceae, Lamiaceae, Rutaceae [32], Euphorbiaceae, Leguminosae, and Solanaceae [8].

In this study, we tested the following hypotheses: (i) the aqueous extracts of native plants from the Brazilian savannah (*Alibertia* spp.) affect the development of *P*. *xylostella*; (ii) there are morphological changes in insects when in contact with the aqueous extracts; and (iii) *Alibertia* spp. presents a great potential as insecticidal plant. Given the few studies and potential of the members of the Rubiaceae family, the present study investigated the effect of aqueous extracts of *Alibertia edulis*, *Alibertia intermedia*, and *Alibertia sessilis* on the biological cycle of *P. xylostella*.

## 2. Materials and Methods

### 2.1. Rearing of Plutella xylostella

The larvae and pupae of *P. xylostella* were collected from cabbage plantation areas in the cities of Dourados (22°13′16′′ S 54°48′20′′ W) and Itaporã (22°45′6′′ S 54°47′20′′ W), both in the state of Mato Grosso do Sul. The collected *P. xylostella* were reared at the Insect-Plant Interaction Laboratory of the Faculty of Biological and Environmental Sciences at the Federal University of Grande Dourados (UFGD), Mato Grosso do Sul, Brazil. Individuals were maintained under constant temperature (25 ± 2 °C) and relative humidity (55% ± 5%).

The pupae were placed in transparent plastic cages until the adults emerged. Adults were fed with a 10% honey solution, and were provided with an oviposition substrate of cabbage discs measuring 8 cm in diameter placed on moist filter paper.

After oviposition, the leaves holding the eggs were placed in sterile plastic containers measuring 30 cm in length, 15 cm in width, and 12 cm in height. Once hatched, the larvae remained in this container until reaching the pupal stage. The larvae were fed on leaves of organic cabbage (*Brassica oleracea* var. *acephala* DC) that were first treated with 5% sodium hypochlorite solution and then washed in tap water.

**Figure 1:**
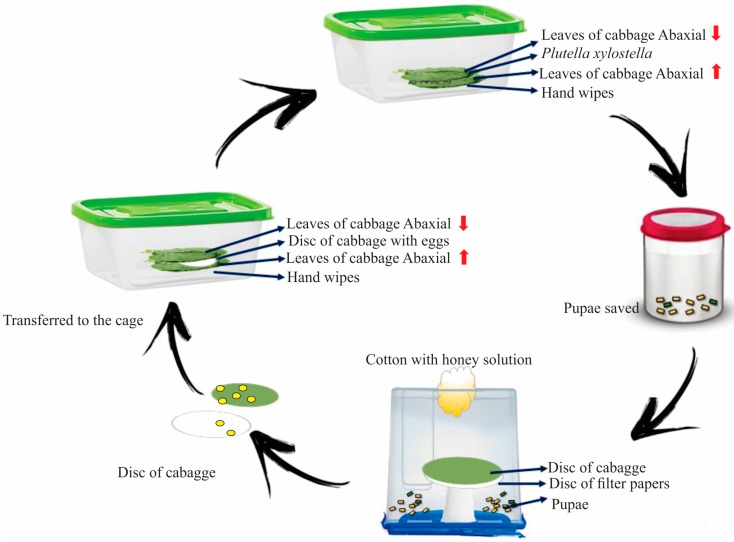
A schematic representation of the methodology used for rearing *Plutella xylostella*, by Matias da Silva et al. [34].

Young cabbage leaves, located in the third and fourth node, were collected from plants that had 90 days of growth in the organic garden of the UFGD. Healthy cabbage leaves were placed with the adaxial face against the plastic container. The larvae were placed on the abaxial face, and then covered with another cabbage leaf that was oriented with the abaxial face covering them. This procedure was repeated daily or when the leaves wilted, whichever occurred first, and continued until pupae formation [33] (Figure 1).

Expanded leaves of *Alibertia edulis*, *Alibertia intermedia*, and *Alibertia sessilis* were collected from Coqueiro farm in the municipality of Dourados, MS (22°12′ S 54°54′ W; 452 m altitude) from 7 a.m. to 9 a.m. Authorization for collection of botanical material was granted by the Brazilian National Research Council (CNPq)/Council of Genetic Heritage Management (CGEN/MMA, number 010220/2015-1).

The plant species were identified by a specialist in the laboratory of Applied Botany, and exsiccated samples were deposited at the Herbarium of the Federal University of Grande Dourados, Mato Grosso do Sul, Brazil, with the following registration numbers: *Alibertia edulis*: 5409; *Alibertia intermedia*: 5408; and *Alibertia sessilis*: 5410.

### 2.2. Preparation of Aqueous Extracts

The leaves of *A. edulis*, *A. intermedia*, and *A. sessilis* were dried in a forced circulating air oven for three days at a maximum temperature of 40 °C (±1 °C). Thereafter, they were ground in a mill to obtain a fine powder.

Aqueous extracts of *A. edulis*, *A. intermedia*, and *A. sessilis* were prepared by maceration. In total, 10 g of the aforementioned powder was manually shaken with 100 mL of distilled water. The extracts were cooled at 10 °C for 24 h and, thereafter, filtered through voile fabric, yielding extracts with a 10% (*w*/*v*) concentration.

### 2.3. Bioactivity of A. edulis, A. intermedia, and A. sessilis Aqueous Extracts against P. xylostella

To assess the bioactivity of the extracts of *A. edulis*, *A. intermedia*, and *A. sessilis*, cabbage leaf discs (*B. oleracea* var. *acephala*) of 8 cm in diameter were immersed in the aqueous extracts (10 g/mL) for 1 min. A control was also prepared by immersing identical discs in distilled water. After immersion, the discs were placed on filter paper at room temperature to remove excess moisture, and subsequently transferred to Petri dishes. A single newly-hatched (0–24 h) *P. xylostella* larva was placed in each Petri dish, as determined by the cabbage disc area. The tests were conducted at a temperature of 25 ± 2 °C, a RH of 55% ± 5%, and a photoperiod of 12 h.

To determine larval stage duration, the larvae were monitored continuously until they reached the pupal stage. Since the larvae remained in the parenchyma of the first leaf, the first mortality assessment was performed 48 h after confinement. This assessment consisted of counting the number of dead individuals and replacing cabbage leaf discs. Subsequent evaluations were conducted daily and leaf discs were replaced every 24 h with treated discs (immersed in an aqueous extract (10 g/mL) for 1 min). Pupal survival was determined by isolating individual pupae in 24 or 96 well ELISA plates (Biomerica, Irvine, CA, USA). The pupae were weighed 24 h thereafter and observed.

To evaluate the reproductive stage, eight pairs (*n* = 16) from each treatment, with the exception of *A. sessilis* (two pairs, *n* = 4), were maintained separately in plastic cages containing cabbage leaf discs as oviposition substrates. The fecundity was evaluated daily and larval hatching was observed.

**Figure 2:**
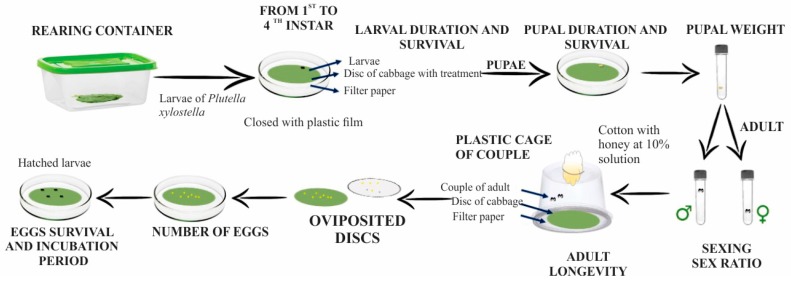
A schematic representation of the methodology used for evaluation of biological parameters, by Matias da Silva et al. [34].

We evaluated the following biological parameters: duration and survival of larval and pupal stages, pupal weight (Bel Mark Analytical Balance, BEL Engineering, Monza, MB, Italy), female and male longevity, sex ratio (female/(female + male), fecundity (total number of eggs deposited throughout life), oviposition (oviposition period (days) = the period between the first and last laying), incubation period (incubation period (days) = the period between egg laying and hatching of larvae), and egg survival (Figure 2).

In addition, individuals with deformities were assessed with respect to the shape and color of the pupae and adult wing shape.

The experimental design was completely randomized, with each treatment consisting of 10 replications of five sub-samples for a total of 50 larvae per treatment. To evaluate the longevity of adult males and females, fecundity, and egg survival of *P. xylostella*, the data were analyzed in a completely randomized design with different repetition number. Data were processed through arcsine transformation, with initial values for larval and pupal survival first converted by √x/100 and initial values for larval and pupal duration, male and female longevity, and fecundity converted by √x + 0.5. The results were submitted to analysis of variance and means were compared with the Tukey test.

### 2.4. Identification of Compounds by High-Performance Liquid Chromatography (HPLC)

The standards and aqueous extracts were analyzed using a Shimadzu LC-6 system, equipped with a diode array detector reading at 200–600 nm, a binary pump, Lab Solution software, a reverse phase C-18 column (25 cm × 4.6 mm × 5 µm), and a pre-column (2.5 cm × 3 mm) of the same phase. The mobile phase consisted of 6% acetic acid in water with 2 mM sodium acetate solution (eluent A) and acetonitrile (eluent B). The analyses were performed using the following gradient elution system: 0 min, 5% B; 30 min, 15% B; 35 min, 30% B; 40 min, 50% B; 45 min, 100% B. The analysis time was 45 min, the flow stream of the pump was 1 mL/min, and the injected volume was 10 µL of extract at a concentration of 100 µg/mL.

The compounds in the samples were identified and quantified by comparing the retention times of the samples with the retention times of pure commercial standards (Sigma-Aldrich, Saint Louis, MO, USA). Standards of caffeic acid, ferrulic acid, *p*-coumaric acid, benzoic acid, cinnamic acid, rutin, quercetin, luteolin, and apigenin (Sigma, ≥97% purity) were prepared at an initial concentration of 100 µg/mL. The concentrations of compounds were determined by external calibration after preparing appropriate dilutions in the range 0.01–10 µg/mL.

## 3. Results

### 3.1. Effect of the Extracts on the Development and Reproduction of P. xylostella

The larvae that were fed on leaves treated with *A. edulis* extract exhibited a reduction in larval period of up to 1.14 days compared with the control (F = 122.24; DF = 3; *p* ≤ 0.001). The extracts of *A. intermedia* and *A. sessilis* caused higher mortality, but prolonged the duration of the larval phase by 3.11 and 2.35 days, respectively, compared with the control (Table 1). The action of *A. intermedia* and *A. sessilis* prevented several larvae from reaching the pupal stage.

Reductions of 43.59% (*n* = 50) and 50.2% (*n* = 50) were observed in the larval survival of *A. intermedia* and *A. sessilis* treated larvae, respectively, when compared with the control. Neither the *A. edulis* extract nor the control promoted significant mortality, with larval survivals greater than 92% being recorded (*n* = 50) (Table 1).

For *A. edulis* extract treatments and control, the pupal duration was 5.67 and 6.08 days, respectively, showing no significant difference. However, *A. intermedia* and *A. sessilis* extracts caused a significant reduction in comparison with the control and *A. edulis*, with values of 3.45 and 2.36 days (F = 13.44; *p* ≤ 0.00003), respectively. For pupal survival, there was a higher mortality in treatments with the extracts of *A. sessilis*, with an adult emergence of 37.19% (F = 7.93; *p* ≤ 0.00055) (Table 1). Although aqueous extracts of *A. intermedia* and *A. sessilis* extended the larval duration compared with the control, this was not reflected by any increase in pupal weight. Specifically, *A. sessilis* (3.46 mg) and *A. intermedia* (3.88 mg) weights were considerably lower than that of the control (5.26 mg) (Table 1). The extracts that caused a reduction in pupal weight exerted higher mortalities in this stage. There was no significant difference in sex ratio between treatments (F = 0.7659; *p* ≤ 0.52338).

In the adult stage, there was no significant difference between treatments for female longevity (F = 1.405; *p* = 0.267); however, males exposed to *A. edulis* showed an increase in longevity (19.37 days) (F = 6.889; *p* = 0.002) (Table 2).

The lowest fecundity was observed in the treatment with *A. intermedia* (86.69) and *A. sessilis* (95.38) (F = 6.7241; *p* ≤ 0.00248). Treatment with all the extracts resulted in a reduction in the number of surviving eggs, with *A. intermedia* (66.24) treatment resulting in the lowest number of hatched larvae (Table 2).

The incubation period for all treatments averaged 3.25 days, with no significant difference among treatments. The oviposition period averaged three days for all treatments.

**Figure 3:**
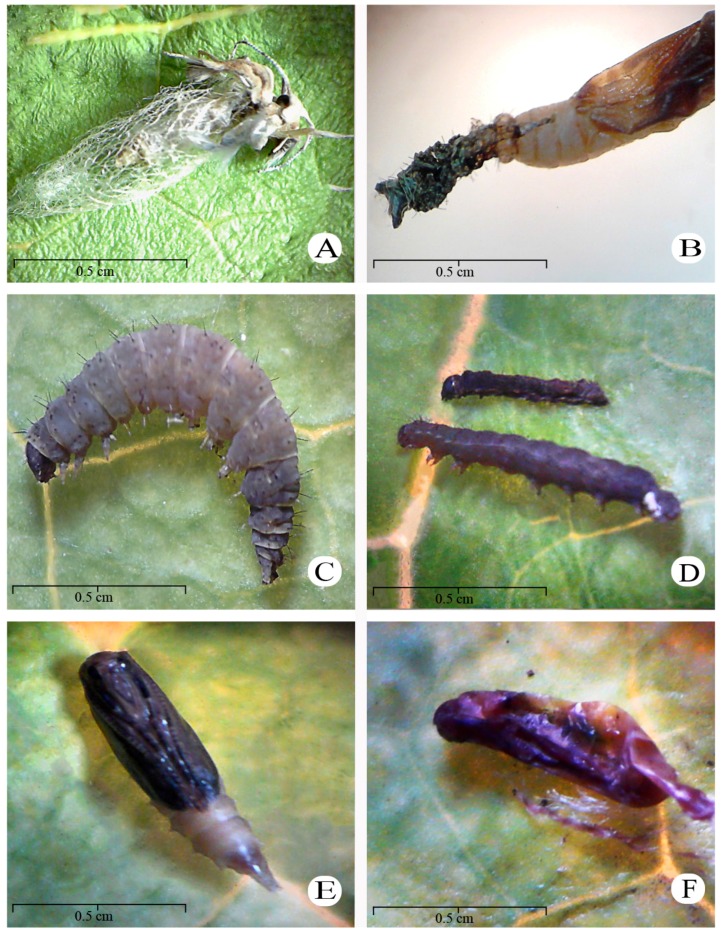
Deformities were observed in adults (**A**), pupae (**B**,**E**,**F**), and larvae (**C**,**D**) of *Plutella xylostella*. (**A**,**D**,**F**) show the effects of *Alibertia sessilis*; (**B**,**C**,**E**) show the effects of *Alibertia intermedia*.

Morphological changes were observed in *P. xylostella* when treated with aqueous extracts of *A. sessilis* and *A. intermedia* (Figure 3A–F). The *A. sessilis*-treated adults had atrophied wings (Figure 3A), whereas pupae had flaccid bodies (Figure 3F) and larvae had a dark color and appeared to be rotting (Figure 3D). Furthermore, individuals exposed to *A. intermedia* died at the pupal stage (Figure 3B or Figure 3E), whereas larvae exhibited a similar dark color (Figure 3C).

### 3.2. Compounds in Aqueous Extracts

**Figure 4:**
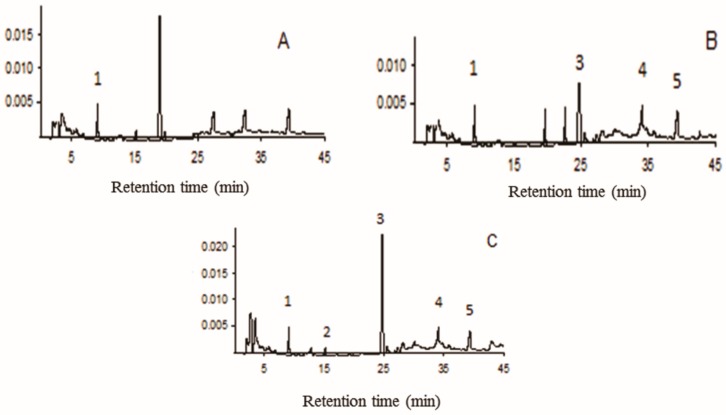
Chromatograms obtained from aqueous extract samples of (**A**) *Alibertia edulis*, (**B**) *Alibertia intermedia*, and (**C**) *Alibertia sessilis* leaves.

Caffeic acid was the only analyzed compound that was present in all the samples examined, whereas *p*-coumaric acid was present only in *A. sessilis*. In addition, rutin, quercetin, and luteolin were present in both *A. intermedia* and *A. sessilis*. *A. sessilis* was the only species that contained all the compounds tested (Figure 4). Table 3 shows the contents of compounds in the samples.

## 4. Discussion

The Rubiaceae species used in this study showed reasonable results regarding their ability to control *P. xylostella* populations. Many previous studies on insects have indicated the antibiosis effects of the substances identified in the extracts of the present study. These substances inhibit food consumption and the morphological and physiological transformations of the pupal stage, which require intense biochemical activity [35,36,37,38,39]. In this study, substances acted during insect metamorphosis—some individuals died between the larval and pupal stages, whereas others emerged as adults with malformed wings or other body parts.

Caffeic acid was found in all three plant species analyzed. Studies have shown the deleterious effect of caffeic acid on larvae of *Helicoverpa zea* (Boddie, 1850) (Lepidoptera: Noctuidae). This compound has potential action in lipid peroxidation and the oxidation of proteins and free iron [38], which could explain the different effects on larval growth and development. Since this was the only compound present in all the extracts analyzed, we believe that it might induce the deformations observed by acting synergistically with other compounds. In addition, other studies have shown that caffeic acid is one of the most efficient feed deterrent compounds against insects [40]. In the great majority of insects, the midgut is the main site of food digestion and assimilation. The feeding habits in Lepidoptera exhibit a different pattern of alkalinity in the midgut, which is related with the physicochemical characteristics of the plant consumed [41]. Changes in the pattern of alkalinity can probably affect the biological and metabolic activities of these insects. Taking this into account, plants containing phenolic compounds, such as caffeic acid, might cause numerous effects on these herbivores, including toxicity [42].

*p*-Coumaric acid, which was observed only in *A. sessilis,* is described as being responsible for the resistance of corn plants against *Sesamia nonagrioides* (Lepidoptera: Noctuidae) [43] and *Prostephunus truncatus* (Horn) (Coleoptera: Bostrichidae) [44]. However, in this study, *p*-coumaric was present only in *A. sessilis*, where it might act as a potential enhancer of synergistic interactions [43], reducing the percentage of larval and pupal survival and pupal weight, and decreasing the longevity of *P. xylostella* females below that observed in the control and other treatments.

In this study, we found that rutin was present in *A. intermedia* and *A. sessilis* extracts, and that the concentration is twice as high in the latter. We observed an increased duration of the larval stage, and a reduced pupal stage, pupal weight, and fecundity in *P. xylostella* treated with extracts of *A. intermedia* and *A. sessilis*. Rutin contributes to plant protection due to its anti-feeding action against Lepidoptera [45]. The actions caused by this flavonoid might extend the larval cycle and cause mortality when added to the diet [46,47], reduce growth and larval and pupal weight [48], decrease pupal survival [49], block feeding, and inhibit digestion and the formation of free radicals [42]. In Lepidoptera, rutin negatively affects the growth of *Anticarsia gemmatalis* Hübner (Lepidoptera: Noctuidae), which is due to pre-ingestion effects, as indicated by reduced consumption, and post-ingestion effects, as indicated by low conversion rates of food ingested into biomass and food assimilation [47]. Rutin also negatively affects the biology of *Spodoptera frugiperda* (Smith, 1797) (Lepidoptera: Noctuidae) by increasing the larval period, and reducing larval and pupal weight and pupal survival [49]. We highlight, however, that further studies are needed to understand the synergic interaction between compounds [43] and the isolates of each compound found in the extracts.

The quercetin present in *A. intermedia* and *A. sessilis* influences the biology of insects. While assessing the biology of the soy-caterpillar, *A. gemmatalis*, we observed that quercetin and rutin increased the length of the total life cycle (third instar until the adult stage), and enhanced the mortality rate of caterpillars [50]. The authors observed that by administering quercetin, there was a reduction in the mortality rate and pupal weight; however, the phase duration was not changed. The results showed that the effects of this flavonoid on insects appear from the third or fourth instar, but are more intense in the fifth and sixth instars.

Luteolin is a flavonoid that impedes insect oviposition on leaves [51,52], and this characteristic corroborates the results obtained in the present study with respect *A. intermedia* and *A. sessilis* treatments. In *Acyrthosiphon pisum* Harris (Homoptera: Aphididae), we observed changes in fecundity with different doses of luteolin [53]. Thus, it is possible that this flavonoid is directly related to changes in insect fecundity, reinforcing the hypothesis of a correlation between this compound and fecundity.

We highlight, however, that the roles of these compounds in plant–insect interactions are very variable. Nevertheless, many flavonoids, depending on the dose, do reduce food intake [54,55,56,57,58,59].

We believe that the flavonoids rutin, quercetin, and luteolin present in *A. intermedia* and *A. sessilis* are probably responsible for the changes in *P. xylostella* observed in the present study.

Given these results and the consulted literature, we believe that the observed insect-plant inter-relationships would ensure crop protection. Nevertheless, the responses of insects to phenolic compounds are variable and, thus, warrant further in-depth studies on the relevant mechanisms of action, since many aspects remain unknown.

## 5. Conclusions

The results of this study have contributed to enhancing our understanding of the insecticidal activity of species of *Alibertia* against *P. xylostella*. The species, particularly *A. intermedia* and *A. sessilis,* should, accordingly, be the focus of further in-depth studies for more thorough assessments. However, given that these results were obtained under laboratory conditions, semi-field and field studies should also be conducted, particularly using the substances identified in this study.

## Figures and Tables

**Table 1:** Duration (days) and survival (%) of larval and pupal stages and pupal weight (mg) of *Plutella xylostella* L. treated with *Alibertia edulis*, *Alibertia intermedia*, and *Alibertia sessilis* aqueous extracts.

Treatment	Larval Duration (Days)	Larval Survival (%)	Pupal Duration (Days)	Pupal Survival (%)	Pupal Weight (mg)
Control	5.86 ± 0.21 b * n = 50	96.67 ± 4.3 a *n* = 50	6.08 ± 0.17 a *n* = 46	98.49 ± 4.44 a *n* = 46	5.26 ± 0.3 a *n* = 46
*A. edulis*	4.72 ± 0.15 c *n* = 50	92.46 ± 3.3 a *n* = 50	5.67 ± 0.12 a *n* = 44	97.73 ± 3.59 a *n* = 44	4.61 ± 0.4 ab *n* = 44
*A. intermedia*	8.97 ± 0.19 a *n* = 50	56.41 ± 6.5 b *n* = 50	3.45 ± 0.12 b *n* = 28	96.34 ± 5.83 a *n* = 28	3.88 ± 0.2 bc *n* = 28
*A. sessilis*	8.21 ± 0.13 a *n* = 50	49.80 ± 4.5 b *n* = 50	2.36 ± 0.64 b *n* = 25	37.19 ± 11.7 b *n* = 11	3.46 ± 0.2 c *n* = 11
CV (%)	3.9	21.64	16.4	30.55	20.6

* Means followed by different letters in the same column differ at 5% significance level when compared using the Tukey test; *n* = number of individuals.

**Table 2:** Longevity of adult males and females, fecundity and egg survival (%) of *Plutella xylostella* L. treated with of *Alibertia edulis*, *Alibertia intermedia*, and *Alibertia sessilis* aqueous extracts.

Treatment	Longevity of Males (Days)	Longevity of Females (Days)	Fecundity	Survival of Eggs (%)
Control	13.26 ± 1.41 b * n = 8	12.43 ± 1.19 a *n* = 8	198.08 ± 9.44 a	91.75 ± 1.14 a
*A. edulis*	19.37 ± 1.14 a *n* = 8	12.81± 0.66 a *n* = 8	187.57 ± 17.53 a	71.04 ± 4.07 ab
*A. intermedia*	14.66 ± 0.86 b *n* = 8	12.39 ± 0.90 a *n* = 8	86.69 ± 18.46 b	66.24 ± 9.53 b
*A. sessilis*	10.49 ± 0.50 b *n* = 2	8.97 ± 1.0 a *n* = 2	95.38 ± 6.5 ab	77.41 ± 3.95 ab
CV (%)	10.15	10.1	20.36	21.97

* Means followed by different letters in the same column differ at 5% significance level when compared using the Tukey test; *n* = number of individuals.

**Table 3:** Compounds identified and quantified by HPLC analysis.

Peaks	Compound	Retention Time (min)	Concentration (mg/g ± SD) A	Concentration (mg/g ± SD) B	Concentration (mg/g ± SD) C
1	Caffeic acid	10.1	51.8 ± 0.1	48.9 ± 0.3	47.8 ± 0.2
2	*p*-Coumaric acid	15.2	-	-	10.7 ± 0.4
3	Rutin	24.7	-	88.9 ± 0.3	178.9 ± 0.6
4	Quercetin	34.5	-	25.7 ± 0.1	23.9 ± 0.1
5	Luteolin	39.7	-	32.5 ± 0.2	33.1 ± 0.1

SD = Standard deviation; “-” = absent values.
